# Refugee migration and risk of schizophrenia and other non-affective psychoses: cohort study of 1.3 million people in Sweden

**DOI:** 10.1136/bmj.i1030

**Published:** 2016-03-15

**Authors:** Anna-Clara Hollander, Henrik Dal, Glyn Lewis, Cecilia Magnusson, James B Kirkbride, Christina Dalman

**Affiliations:** 1 Department of Public Health Sciences, Karolinska Institutet, 171 77 Stockholm, Sweden; 2 Centre for Epidemiology and Community Medicine, Stockholm County Council, SE-171 77 Stockholm, Sweden; 3 Division of Psychiatry, University College London, London W1T 7NF, UK

## Abstract

**Objective** To determine whether refugees are at elevated risk of schizophrenia and other non-affective psychotic disorders, relative to non-refugee migrants from similar regions of origin and the Swedish-born population.

**Design** Cohort study of people living in Sweden, born after 1 January 1984 and followed from their 14th birthday or arrival in Sweden, if later, until diagnosis of a non-affective psychotic disorder, emigration, death, or 31 December 2011.

**Setting** Linked Swedish national register data.

**Participants** 1 347 790 people, including people born in Sweden to two Swedish-born parents (1 191 004; 88.4%), refugees (24 123; 1.8%), and non-refugee migrants (132 663; 9.8%) from four major refugee generating regions: the Middle East and north Africa, sub-Saharan Africa, Asia, and Eastern Europe and Russia.

**Main outcome measures** Cox regression analysis was used to estimate adjusted hazard ratios for non-affective psychotic disorders by refugee status and region of origin, controlling for age at risk, sex, disposable income, and population density.

**Results** 3704 cases of non-affective psychotic disorder were identified during 8.9 million person years of follow-up. The crude incidence rate was 38.5 (95% confidence interval 37.2 to 39.9) per 100 000 person years in the Swedish-born population, 80.4 (72.7 to 88.9) per 100 000 person years in non-refugee migrants, and 126.4 (103.1 to 154.8) per 100 000 person years in refugees. Refugees were at increased risk of psychosis compared with both the Swedish-born population (adjusted hazard ratio 2.9, 95% confidence interval 2.3 to 3.6) and non-refugee migrants (1.7, 1.3 to 2.1) after adjustment for confounders. The increased rate in refugees compared with non-refugee migrants was more pronounced in men (likelihood ratio test for interaction χ^2^ (df=2) z=13.5; P=0.001) and was present for refugees from all regions except sub-Saharan Africa. Both refugees and non-refugee migrants from sub-Saharan Africa had similarly high rates relative to the Swedish-born population.

**Conclusions** Refugees face an increased risk of schizophrenia and other non-affective psychotic disorders compared with non-refugee migrants from similar regions of origin and the native-born Swedish population. Clinicians and health service planners in refugee receiving countries should be aware of a raised risk of psychosis in addition to other mental and physical health inequalities experienced by refugees.

## Introduction

Schizophrenia and other psychotic disorders lead to lifelong health and social adversities, culminating in a reduction in life expectancy of 10-25 years.^1^ Immigrants and their descendants are, on average, 2.5 times more likely to have a psychotic disorder than the majority ethnic group in a given setting,^2^ ^3^ although the exact risk varies by ethnicity and setting. For example, in Europe, incidence rates for people of black Caribbean or African descent are approximately five times higher than those for the white European population.^2^ ^4^ These marked differences persist after adjustment for age, sex, and socioeconomic position,^5^ are maintained in the descendants of first generation migrants,^2^ and do not seem to be attributable to higher incidence rates in people’s country of origin or selective migration.^6^ ^7^ ^8^ ^9^ Possible explanations centre on various social determinants of health, including severe or repeated exposure to psychosocial adversities such as trauma, abuse, socioeconomic disadvantage, discrimination, and social isolation. If this is the case, people granted refugee status may be particularly vulnerable to psychosis, given their increased likelihood of having experienced conflict, persecution, violence, or other forms of psychosocial adversity.^10^ ^11^

Although refugees have more mental health problems than their non-refugee counterparts,^11^ ^12^ including post-traumatic stress disorder and common mental disorders,^13^ ^14^ little is known about the risk of psychosis in refugees. One previous longitudinal study from Denmark observed that refugees were at elevated risk of psychosis compared with the native-born Danish population.^15^ However, the risk in refugees was not compared with that in other non-refugee migrants (henceforth referred to as migrants), who are known to be at increased risk,^16^ making attribution of this excess directly to a refugee effect impossible. More recently, a Canadian cohort study found that refugees had a modestly increased risk of schizophrenia compared with other migrants,^17^ but neither group was at elevated risk compared with an ethnically diverse Canadian-born background population, making this finding difficult to interpret and contrary to a large literature on immigration and psychosis.^2^

Here, we clarify the risk of non-affective psychotic disorders, including schizophrenia, in refugees compared with other migrants and the native-born Swedish population in a national population based cohort of 1.3 million people. Sweden has a total population size of 9.7 million inhabitants, of whom 1.6 million were born abroad. In 2011 refugees constituted 12% of the total immigrant population. Sweden experienced high levels of labour immigration between 1940 and 1970, followed by substantial refugee immigration.^18^ On a per capita basis, Sweden grants more refugee applications than any other high income country,^19^ which, combined with national linked register data, makes it an excellent setting in which to conduct this research. We hypothesised that refugees would have a higher risk of non-affective psychotic disorders than migrants and that risk for both groups would be elevated compared with the Swedish-born population. We also hypothesised that the risk in refugees compared with migrants would vary by region of origin, given putative differences in the pre-migratory experiences of migrants from different regions and differences in how they might adjust to a new society.

## Methods

### Study design and population

We established a retrospective cohort of 1 347 790 people born after 1 January 1984, who were born in Sweden to two Swedish-born parents (n=1 191 004; 88.4%) or were refugees (n=24 123; 1.8%) or non-refugee first generation migrants (n=132 663; 9.8%) granted residency in Sweden. To permit valid comparisons between refugees and migrants, we restricted the immigrant sample to people born in geographical regions with at least 1000 refugees in our cohort (see below). We excluded people without an official residence permit in Sweden—that is, undocumented migrants or people with an official asylum decision pending. We followed participants from their 14th birthday, or date of arrival in Sweden if later, until diagnosis of an ICD-10 (international classification of diseases, 10th revision) non-affective psychotic disorder (F20-29), emigration, death, or 31 December 2011, whichever was sooner. We could not include people who immigrated to Sweden before 1 January 1998 (n=53 855), because refugee status was not sufficiently recorded in the Swedish national registers before this date. We also excluded 812 (0.06%) participants with missing data on municipality of residence in Sweden at cohort entry, needed for estimation of urban residency as a covariate (see below). Excluded participants did not differ from immigrants included in the cohort by sex (51.0% (27 471/53 855) versus 50.7% (79 863/157 531) men; χ^2^ P=0.21) but had a higher disposable income (11.0% (5924/53 855) versus 5.4% (8533/157 531) were in the highest income quarter; χ^2^ P<0.001) and were more likely to come from the former Yugoslavia (32.4% (17 457/53 855) versus 8.4% (13 275/157 531); χ^2^ P<0.001) than other regions. Crude incidence rates were similar between excluded (77.7 (95% confidence interval 70.4 to 85.8) per 100 000 person years) and included immigrants (86.6 (79.1 to 94.7) per 100 000 person years).

### Data sources

We extracted data from a large, longitudinal database of linked national registers, known as Psychiatry Sweden, which included data on all people officially resident in Sweden after 1 January 1932, linked via a unique personal identity number and anonymised by Statistics Sweden for research purposes. We obtained relevant outcome, exposure, and covariate data from the following registers: the register of the total population to identify cohort participants and obtain basic demographic data (birth date, sex, country of birth); the multi-generation register to link participants to their parents for identification of the native-born Swedish population; the longitudinal integration database for health insurance and labour market studies (LISA) to obtain data on disposable income; the immigration and emigration database (STATIV) to obtain migration and refugee data; the national patient register to obtain outcome data; and the causes of death register for data pertaining to mortality.

### Patient involvement

No patients were involved in setting the research question or the outcome measures, nor were they involved in developing plans for design or implementation of the study. No patients were asked to advise on interpretation or writing up of results. However, we will disseminate the results of our research to agencies responsible for the healthcare of refugee and migrant groups in Sweden.

### Outcome

Our primary outcome was an ICD-10 clinical diagnosis of non-affective psychotic disorder (F20-29), which included schizophrenia (F20) and all other non-affective psychotic disorders (F21-29). We defined cases as cohort participants with a first recorded diagnosis between 1 January 1998 and 31 December 2011 in the national patient register, which records diagnoses following inpatient and outpatient admissions in Sweden (including privately run public healthcare settings). Inpatient records are complete since 1987, and complete recording from outpatient settings began in 2001. We excluded anyone with a recorded diagnosis of non-affective psychotic disorder made before the age of 14 years (n=156).

### Exposures

Our primary exposure was refugee status, defined as refugee, other migrant, or person born in Sweden to two Swedish-born parents, obtained from the STATIV database, which records the reason why a residence permit was granted. Permanent residency for asylum in Sweden is based on the Swedish Migration Agency’s definition of refugee status,^18^ made in accordance with Swedish law and the UN Refugee Convention, as someone who, “owing to a well-founded fear of being persecuted . . . is unable to, or owing to such fear, is unwilling to avail himself of the protection of that country.”^20^ All other immigrants granted official residency were classified as migrants. We identified people born in Sweden to two Swedish-born parents (henceforth the “Swedish-born” group) via linkage to the multi-generation register.

As a secondary exposure, we classified people according to region of origin, as defined by country of birth. Although Statistics Sweden records data on specific country of birth, information is released for research purposes according to 13 larger geographical regions to ensure confidentiality. From this variable, we derived a broader region of origin variable for analysis, which included Sweden (Swedish-born only) and four other regions from which at least 1000 refugees in our cohort originated—sub-Saharan Africa, Asia, eastern Europe and Russia, and the Middle East and north Africa (see supplementary table A).

### Confounders

We included age at risk and sex as two a priori confounder variables in all analyses. We also included individual disposable income in Sweden and population density at cohort entry as covariates, to adjust for possible differences between refugees, migrants, and the Swedish-born population.

We defined disposable income as annual disposable income, based on total family income from all registered sources, including wages, welfare benefits, other social subsidies, and pensions. Statistics Sweden estimated individual disposable income, weighting total family income according to household size and composition, with younger children given lower weights than older household members. We measured disposable income at the earliest point during follow-up (available in LISA at 16 years old or arrival in Sweden, if later). To account for inflation, we categorised individual disposable income into quarters, relative to all other cohort members assigned a disposable income score in the same year.

We defined urban residency according to the population density of each participant’s municipality of residence at cohort entry, expressed as the total population per square kilometre (ppkm^2^). Sweden consists of 290 municipalities (median population density 26.3 (interquartile range 12.2-75.7) ppkm^2^). For descriptive purposes, we classified participants into three population density categories: 0-26.2 ppkm^2^ (very rural areas, below Swedish median), 26.3-260 ppkm^2^ (rural and semi-rural areas), and 260.1-4617.2 ppkm^2^ (metropolitan, suburban, and urban areas). To adjust more effectively for population density, we used a continuous measure in our analyses, first transformed on to the natural logarithm scale to account for its positive skewed distribution across municipalities.

### Statistical analyses

We recorded basic descriptive statistics and crude incidence rates for refugees, migrants, and the Swedish-born group. Next, we fitted Cox proportional hazard models to estimate hazard ratios and 95% confidence intervals according to each exposure variable. Follow-up time was based on the earliest date of entry into the risk period (date of 14th birthday or, for all immigrants older than 14 years on arrival, date of immigration) until exit from the cohort. We modelled age at risk as a time varying covariate, using Lexis expansion to stratify each participant into N observations, taking into account differing ages at risk over the follow-up period (14-16, 17-19, 20-22, 23-25, 26-27; N_max_=5).

We initially examined the effect of refugee status on risk of non-affective psychotic disorder, after adjustment for age at risk, sex, and their interaction, if statistically significant. In a second adjustment, we added disposable income and population density. We tested whether the relation between refugee status and non-affective disorder differed between men and women by fitting an interaction term between refugee status and sex, with results presented separately for men and women, where appropriate. We repeated these analyses for our secondary exposure variable, region of origin. Next, to determine whether risk of non-affective psychotic disorder in refugees relative to migrants differed by region of origin, we fitted a Cox regression model to a subset of the cohort, excluding the Swedish-born group who did not contribute information to these analyses. Given the small sample of female refugees diagnosed as having psychosis (n=27), we did these analyses for both sexes combined and, separately, for men only. We assessed all statistical interactions by using likelihood ratio tests against a model without the relevant interaction term.

To minimise the possibility that any immigrants diagnosed as having non-affective psychotic disorder may have been prevalent (that is, existing) cases on arrival in Sweden, we did sensitivity analyses on all models, excluding any refugee or non-refugee migrant given a diagnosis within 12 months of immigration. Finally, we checked our main models (via likelihood ratio tests) for departure from proportional hazards. We used Stata v13 to analyse the data.

## Results

We identified 3704 cases during more than 8.9 million person years of follow-up (table 1). Median age at first diagnosis in the Swedish-born population was 20.1 (interquartile range 18.3-22.3) years, younger than for refugees (21.0 (19.2-23.7) years; Mann-Whitney P<0.001) and non-refugees (20.9 (18.7-23.6) years; P<0.001), for whom age at first diagnosis was similar (P=0.30). Following arrival in Sweden, time to first diagnosis was shorter for refugees (median 2.8 (0.7-5.6) years) than for migrants (3.9 (1.2-7.0) years; Mann-Whitney P=0.02).

**Table 1 tbl1:** Cohort characteristics by migrant status—refugees, non-refugee migrants, and Swedish-born population. Values are numbers (percentages)

Characteristics	Swedish-born population			Non-refugee migrants			Refugee migrants	
	Cases (n=3232)	Person years* (n=8 384 891)		Cases (n=379)	Person years* (n=471 308)		Cases (n=93)	Person years* (n=73 604)
Sex:								
Men	1778 (55.0)	4 310 990 (51.4)		234 (62)	232 118 (49.2)		66 (71)	41 069 (55.8)
Women	1454 (45.0)	4 073 901 (48.6)		145 (38)	239 190 (50.8)		27 (29)	32 535 (44.2)
Birth year:								
1984-86	1279 (39.6)	2 928 401 (34.9)		175 (46)	185 052 (39.3)		35 (38)	23 820 (32.4)
1987-89	1111 (34.4)	2 510 835 (29.9)		107 (28)	125 770 (26.7)		28 (30)	19 093 (25.9)
1990-92	649 (20.1)	1 896 903 (22.6)		74 (20)	91 965 (19.5)		22 (24)	16 837 (22.9)
1993-95	174 (5.4)	903 840 (10.8)		19 (5)	56 237 (11.9)		8 (9)	11 728 (15.9)
1996-97	19 (0.6)	144 911 (1.7)		4 (1)	12 283 (2.6)		0 (0)	2127 (2.9)
Region of origin:								
Sweden	3232 (100.0)	8 345 891 (100.0)		-	-		-	-
Sub-Saharan Africa	-	-		111 (29)	59 447 (12.6)		31 (33)	18 670 (25.4)
Asia	-	-		66 (17)	105 647 (22.4)		15 (16)	12 929 (17.6)
Eastern Europe	-	-		80 (21)	134 094 (28.5)		7 (8)	6546 (8.9)
Middle East	-	-		122 (32)	172 120 (36.5)		40 (43)	35 459 (48.2)
Income:								
Lowest quarter	1156 (35.8)	2 161 330 (25.8)		264 (70)	339 062 (71.9)		63 (68)	51 953 (70.6)
Second quarter	830 (25.7)	2 185 386 (26.1)		52 (14)	63 153 (13.4)		12 (13)	10 486 (14.2)
Third quarter	679 (21.0)	2 073 841 (24.7)		45 (12)	35 919 (7.6)		13 (14)	6768 (9.2)
Highest quarter	567 (17.5)	1 964 334 (23.4)		18 (5)	33 174 (7.0)		5 (5)	4398 (6.0)
Population density†:								
0-26.2	875 (27.1)	2 303 728 (27.5)		50 (1)	55 129 (11.7)		25 (27)	21 746 (29.5)
26.3-260	1698 (52.5)	4 472 698 (53.3)		168 (44)	216 155 (45.9)		49 (53)	35 031 (47.6)
260.1-4617.2	659 (20.4)	1 608 466 (19.2)		161 (42)	200 024 (42.4)		19 (20)	16 827 (22.9)

*Rounded to nearest integer.

†People per km^2^.

The crude incidence rate of non-affective psychotic disorders was 38.5 (95% confidence interval 37.2 to 39.9) per 100 000 person years in the Swedish-born population, 80.4 (72.7 to 88.9) per 100 000 person years in migrants, and 126.4 (103.1 to 154.8) per 100 000 person years in refugees. This corresponded to an absolute rate difference of 45.9 (19.0 to 72.9) per 100 000 person years in refugees compared with migrants, in addition to an extra 41.9 (33.7 to 50.1) cases per 100 000 person years in migrants compared with the Swedish-born population. Compared with the Swedish-born population, hazard ratios were 2.90 (95% confidence interval 2.31 to 3.64) in refugees and 1.75 (1.51 to 2.02) in migrants, after adjustment for age, sex, their interaction, disposable income, and population density (table 2). Refugees were 1.66 (1.32 to 2.09) times more likely to be diagnosed as having non-affective psychotic disorders than were migrants. These associations were more pronounced in men than women (likelihood ratio test P for interaction=0.001; table 2 and fig 1).

**Table 2 tbl2:** Risk of non-affective psychoses by migrant status after adjustment for confounders. Values are hazard ratios (95% CIs)

Category	All			Men			Women	
	Model 1	Model 2		Model 1	Model 2		Model 1	Model 2
**Swedish-born as reference**								
Non-refugee migrant	2.28 (1.99 to 2.62)	1.75 (1.51 to 2.02)		2.61 (2.22 to 3.07)	2.01 (1.70 to 2.38)		1.91 (1.58 to 2.31)	1.44 (1.19 to 1.76)
Refugee migrant	3.61 (2.87 to 4.53)	2.90 (2.31 to 3.64)		4.28 (3.28 to 5.58)	3.49 (2.67 to 4.55)		2.65 (1.80 to 3.92)	2.07 (1.40 to 3.06)
**Non-refugee migrant as reference**								
Refugee migrant	1.58 (1.26 to 1.99)	1.66 (1.32 to 2.09)		1.64 (1.25 to 2.15)	1.74 (1.32 to 2.28)		1.39 (0.92 to 2.10)	1.43 (0.95 to 2.16)

Model 1 was adjusted for age at risk, sex, and their interaction. Model 2 was also adjusted for disposable income and population density. A likelihood ratio test confirmed statistical interaction between sex and age at risk in model 1 (χ^2^ (df=4) 71.5; P<0.001) and model 2 (χ^2^ (df=4) 73.0; P<0.001), as well as between sex and refugee status in model 1 (χ^2^ (df=2) 11.7; P=0.003) and model 2 (χ^2^ (df=2) 13.5; P=0.001). Hazard ratios by refugee status are therefore presented separately for men and women.

**Figure f1:**
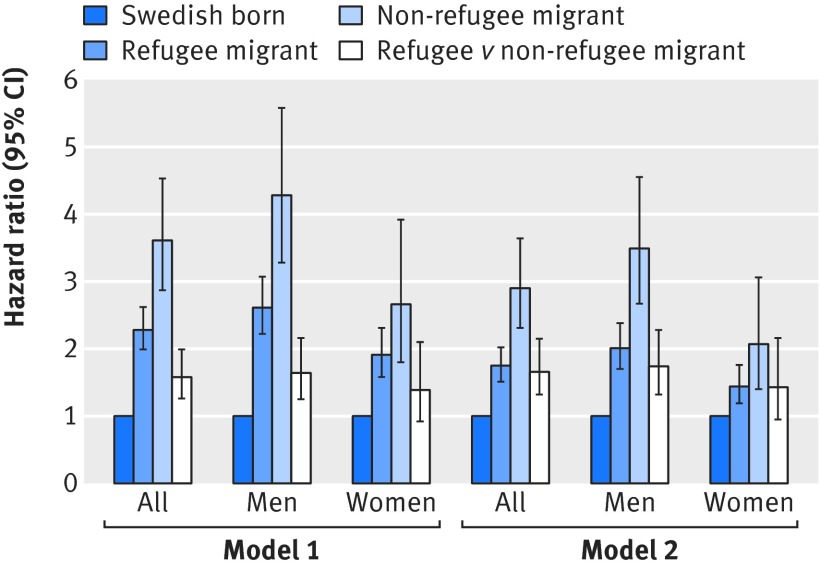
**Fig 1** Hazard ratios for schizophrenia and other non-affective psychotic disorders by refugee status and sex. Model 1 was adjusted for age at risk, sex, and their interaction (where appropriate). Model 2 was additionally adjusted for disposable income and population density. Swedish-born group provides reference category, except for fourth (white) bar in each group, which shows hazard ratio for refugees relative to non-refugee migrants. Error bars represent 95% confidence intervals

Taking refugees and migrants together, immigrants from all regions of origin had increased rates of disorder relative to the Swedish-born population, after adjustment for age at risk and sex (supplementary table B). Hazard ratios were most pronounced for all immigrants from sub-Saharan Africa (hazard ratio 5.23, 4.32 to 6.34), which was also observed for both men (6.68, 5.33 to 8.37) and women (3.64, 2.68 to 4.94) separately. These patterns persisted after adjustment for disposable income and population density, ranging from 1.41 (1.11 to 1.78) in people from eastern Europe and Russia to 4.10 (3.38 to 4.98) in people from sub-Saharan Africa, relative to the Swedish-born population.

We next investigated whether the elevated rates of non-affective psychotic disorders in refugees compared with migrants differed by region of origin, excluding the Swedish-born population who did not contribute to these analyses. For men and women combined, we found evidence that the rate of non-affective psychosis in refugees compared with migrants varied by region of origin (table 3; likelihood ratio test P=0.05). This finding was even more pronounced in men (likelihood ratio test P=0.007), such that rates of non-affective psychotic disorder were elevated in refugees compared with migrants from all regions of origin, except sub-Saharan Africa (hazard ratio 0.68, 0.40 to 1.16), after adjustment for age at risk, sex, disposable income, and population density (table 3). Male refugees from eastern Europe and Russia were at greatest risk compared with their migrant counterparts (hazard ratio 2.88, 1.22 to 6.82). In general, the rate of psychotic disorders in refugees relative to migrants became smaller as the crude incidence rate in non-refugees from each region of origin increased (table 3). We made no attempt to examine this effect in women, given insufficient numbers of refugees (n=27).

**Table 3 tbl3:** Risk of non-affective psychoses in refugees relative to non-refugees, by region of origin

Category	All			Men	
	Crude incidence rate (95% CI) per 100 000 PYAR	Hazard ratio (95% CI): model 2		Crude incidence rate (95% CI) per 100 000 PYAR	Hazard ratio (95% CI): model 2
Swedish-born	38.5 (37.2 to 39.9)	-		41.2 (39.4 to 43.2)	-
Eastern Europe:					
Non-refugees	59.7 (47.9 to 74.3)	1		62.5 (45.9 to 85.2)	1
Refugees	106.9 (51.0 to 224.3)	1.76 (0.81 to 3.82)		184.1 (82.7 to 409.8)	2.88 (1.22 to 6.82)
Asia:					
Non-refugees	62.5 (49.1 to 79.5)	1		67.0 (48.3 to 92.9)	1
Refugees	116.0 (69.9 to 192.4)	1.78 (1.01 to 3.14)		146.1 (83.0 to 257.3)	2.20 (1.13 to 4.25)
Middle East and north Africa:					
Non-refugees	70.9 (59.4 to 84.6)	1		94.4 (75.9 to 117.4)	1
Refugees	112.8 (82.7 to 153.8)	1.56 (1.08 to 2.23)		143.5 (100.3 to 205.2)	1.55 (1.01 to 2.36)
Sub-Saharan Africa:					
Non-refugees	186.7 (155.0 to 224.9)	1		269.0 (215.1 to 336.3)	1
Refugees	166.0 (116.8 to 236.1)	0.81 (0.54 to 1.23)		207.1 (130.5 to 328.8)	0.68 (0.40 to 1.16)

Estimates from model 1 and model 2 were similar; only data from model 2, adjusted for age at risk, sex, their interaction (for both sexes combined), disposable income, and population density, are reported. Likelihood ratio test χ^2^ (df=3) and P values, for statistical interaction between refugee status and region of origin were 8.0 and 0.05 for full sample and 12.0 and 0.007 in analysis restricted to men. Given small number of refugee women with outcome (n=27), no attempt was made to inspect risk by region of origin separately for women.

PYAR=person years at-risk.

Sensitivity analyses excluding potentially prevalent cases among immigrants did not appreciably alter estimates of associations for our main exposures (supplementary tables C and D). The assumption of proportional hazards was not violated (P=0.84 and P=0.13 for analyses of refugee status and region of origin, respectively).

## Discussion

In this cohort study, we found that refugees granted asylum in a high income setting were, on average, 66% more likely to develop schizophrenia or another non-affective psychotic disorder than non-refugee migrants from the same regions of origin and up to 3.6 times more likely to do so than the Swedish-born population.

### Strengths and weaknesses of study

This study has several methodological strengths. It was based on a large, national population based cohort of more than 1.3 million people, followed for more than 8.9 million person years by using linked Swedish register data. This research has not previously been possible owing to a lack of information on the reason for migration in official Swedish registers; one earlier attempt to investigate this question in Sweden could not distinguish between refugees and non-refugees from the same region.^21^ Swedish register data are known to be reliable for research purposes,^22^ ^23^ and diagnosis of psychotic disorders recorded in the national patient register has good validity and positive predictive value.^24^ ^25^ ^26^ This register is highly complete, recording all psychiatric contacts from inpatient settings from 1987 onwards and from outpatient settings since 2001. Although this may have led to slight under-ascertainment from outpatient settings between 1998 and 2000, we have no reason to believe that this would have introduced differential bias by refugee status or region of origin. We cannot exclude the possibility that we underestimated the true incidence of non-affective psychoses in Sweden, particularly for certain groups, such as recent immigrants or refugees, who may have been unfamiliar with the Swedish healthcare system, have faced greater language barriers, or had poor health literacy.^27^ If these accessibility factors differed according to sex, the true incidence among migrant and refugee women may have been underestimated in the Swedish patient register, making our hazard ratios conservative.

Sensitivity analyses suggested that our results were not attributable to prevalent cases among refugees and migrants. In our study, migrants and refugees from sub-Saharan Africa were at increased risk of having a psychotic disorder, compared with the Swedish-born group. This finding is consistent with many other European and worldwide studies.^2^ Although diagnostic bias has been proposed to explain excess rates of psychotic disorders observed in ethnic minorities,^8^ little evidence supports this possibility in general.^28^ Studies in which psychiatrists were blinded to participants’ ethnicity during the diagnostic process have confirmed rates of psychotic disorders in ethnic minority groups,^29^ including people of black Caribbean and black African origin. In Sweden, by law, interpreters have to aid clinical consultations when necessary. Furthermore, any diagnostic biases are less likely to have accounted for observed differences in risk between refugees and migrants from the same regions of origin observed in our study. Refugees are also at elevated risk of post-traumatic stress disorder,^13^ which can present with psychotic features; however, our findings are unlikely to be attributable to misdiagnosed cases of this disorder among refugees, as it often presents comorbidly in people exposed to potentially traumatic events and experiences.^30^

We were unable to include immigrants who arrived in Sweden before 1998 in our study, because data on refugee status were unavailable before that year. These groups were more likely to come from the former Yugoslavia, reflecting geopolitical conflicts of the time. This may have reduced our power to detect differences between refugees and other migrants from eastern Europe, but we have no reason to believe their exclusion would have otherwise biased our estimates; the crude incidence in this group was comparable to that for included immigrants, despite their higher post-migratory disposable income. Finally, notwithstanding our large cohort size, the number of cases in refugees was small, which limited our power to detect effects in certain groups, most notably women, for whom risk of non-affective psychotic disorders is, on average, half that of men.^31^

As our study was based on routine register data, information on potentially relevant experiences before migration was unavailable. Such pre-migratory experiences remain an important area for future research. Our cohort included migrants and refugees exposed to various humanitarian crises resulting from conflict (such as Iraq, Iran, Afghanistan, the Balkans, central Africa) as well as famine (such as east Africa). Although it is too early to determine whether people currently seeking refuge in Europe following contemporary humanitarian crises (in Syria, Iraq, Afghanistan, parts of north Africa, Kosovo, Albania) would also be at greater risk of psychotic disorder, we assume that our findings will generalise to these groups for two reasons. Firstly, a degree of geographical overlap exists between the regions we included and those generating current humanitarian crises.^32^ Secondly, we presume that exposure to war, persecution, and exposure to other psychosocial adversity would have a universal effect on individual risk of psychosis, independent of other risk factors.

We adjusted for possible differences between refugees, migrants, and the Swedish-born population with regard to age, sex, disposable income, and population density at cohort entry. We did not include other post-migratory markers of potential social disadvantage; such factors may lie on the causal pathway between immigration and risk psychosis, thus making adjustment difficult to interpret. We were unable to examine risk of psychosis in so-called second generation refugees or migrants, because our study population was born after 1984, making their children too young to have entered the risk period for psychosis before the end of our follow-up period in 2011.

### Clinical and public health implications of study

Contemporary humanitarian crises in Europe, the Middle East, north Africa, and central Asia have contributed to more displaced people, asylum seekers, and refugees worldwide than at any time since the second world war.^33^ The severe social, economic, and health inequalities faced by displaced populations arising from these crises are often compounded by national immigration policies and structural constraints in receiving countries. In turn, exposure to these ongoing adversities seems likely to contribute to the increased risk of post-traumatic stress disorder and common mental disorders among refugees.^11^ ^12^ ^13^ Our data highlight further mental health inequalities facing such groups.^34^ Clinicians and service planners in high income settings should be aware of the early signs of psychosis in refugees, for whom median presentation to services after arrival to Sweden was more than a year sooner than for other migrant groups. Just as for the general population, refugees and their families will benefit from timely and early intervention and care, particularly in those exposed to severe psychosocial adversity.

Our findings are consistent with the hypothesis that increased risk of non-affective psychotic disorders among immigrants is due to a higher frequency of exposure to social adversity before migration,^35^ including the effects of war, violence, or persecution. Further studies will be needed to confirm this possibility. Violence experienced by children and adults who flee persecution has been linked to worse subsequent mental health in general.^11^ ^36^ Intriguingly, our study suggested that risk of psychosis in refugees relative to other migrants varied by region of origin in our data. Although this finding needs to be replicated in larger samples, it suggests that in addition to refugee status, context matters. For example, we observed no differences in risk of psychosis between refugees and non-refugee migrants among immigrants from sub-Saharan Africa, perhaps because both groups had highly increased rates of disorder (more than 165 new cases per 100 000 person years).

One parsimonious explanation for this finding is that a larger proportion of sub-Saharan Africa immigrants will have been exposed to deleterious psychosocial adversities before emigration, irrespective of refugee status. By contrast, pre-migratory psychosocial adversities experienced by refugees from eastern Europe and Russia may differ substantially compared with non-refugee migrants from these countries, thus confining excess risk to refugees from such regions. It is also possible that post-migratory factors, such as discrimination, racism, and social exclusion, may explain the high rates of psychotic disorder in migrants and refugees from sub-Saharan Africa, given the absence of a “refugee effect” in this group. Visible minority status may lead to more post-migratory psychosocial adversity. In general population samples, some evidence suggests that perceived discrimination and ethnic density (proximity to one’s own ethnic group) are, respectively, risk and protective factors for psychosis.^37^ ^38^ Although we controlled for income and post-migratory urban residency, we were unable to investigate other post-migratory factors, including racism, discrimination, and ethnic density, in the available data; further exploration of such factors presents an important avenue for future research. Other factors, including difficulties in the asylum process, also warrant further investigation. For example, women seeking asylum are less likely to be granted refugee status than men, given greater structural and cultural barriers in the asylum process.^39^ In our study, such an effect would have led to a higher proportion of women being classified as migrants, which may have partially explained why differences in incidence between female refugees and non-refugees were less pronounced than for their male counterparts. A recent study by Oram et al has further highlighted high levels of severe mental illness faced by trafficked migrants, who represent another vulnerable group of migrants.^40^

### Conclusion

Our study shows that, on average, refugees in a high income setting face substantially elevated rates of schizophrenia and other non-affective psychoses, in addition to the array of other mental, physical, and social inequalities that already disproportionately affect these vulnerable populations. This risk exceeded the well established excess burden of psychosis experienced in immigrant and ethnic minority groups more generally and thus emphasises the need to take the early signs and symptoms of psychosis into account in refugee populations, as part of any clinical mental health service responses to the current global humanitarian crises. More broadly, our findings support the possibility that exposure to psychosocial adversity increases the risk of psychosis.

**What is already known on this topic**

Immigrant populations are at elevated risk of schizophrenia and other non-affective psychotic disorders
Whether refugees have rates of these disorders over and above those typically observed in non-refugee immigrant groups is unclear

**What this study adds**

The incidence rate of a non-affective psychotic disorder was 66% higher among refugees than among non-refugee migrants from similar regions of origin, and nearly three times greater than in the native-born Swedish population
These patterns were apparent for men and women, although they were stronger in men
Refugees from all regions of origin had higher rates of psychotic disorder than non-refugee migrants, except for people from sub-Saharan Africa, for whom rates in both groups were similarly high relative to the Swedish-born population
Clinicians and health service planners should be aware of early signs of psychosis in vulnerable migrant populations, who may benefit from timely and early interventions

## Web Extra

Extra material supplied by the author

## Notes

Contributors: A-CH and CD conceived of the study. A-CH, JK, and CD designed the study and obtained funding. A-CH, CD, and HD acquired the migration data. CD and HD acquired all other cohort data. A-CH, JBK, and HD prepared the data. A-CH, JK, CD, CM, and GL interpreted statistical analyses. HD coordinated the data management, and A-CH wrote the study protocol. A-CH and JBK did the statistical analyses, drafted the data tables, and co-wrote the manuscript. All authors critically revised the paper for important intellectual content and approved the final version. JBK and CD were co-senior authors of the manuscript. A-CH and JBK are the guarantors.

Funding: A-CH is supported by FORTE (grant number 2014-1430; 2014-2678). JBK is supported by a Sir Henry Dale fellowship jointly funded by the Wellcome Trust and the Royal Society (grant number 101272/Z/13/Z). CD is supported by the Swedish Research Council (grant number 523-2010-1052). The funders had no involvement in any aspect of the design of this study, preparation of results, or decision to submit for publication.

Ethical approval: This research has ethical approval as part of Psychiatry Sweden “Psykisk ohälsa, psykiatrisk sjukdom: förekomst och etiologi,” approved by the Stockholm Regional Ethical Review Board (number 2010/1185-31/5).

Data sharing: The statistical code is available from the corresponding author. Under Swedish law and ethical approval, patient level data cannot be made available.

Transparency declaration: The lead authors (the manuscript's guarantors) affirm that the manuscript is an honest, accurate, and transparent account of the study being reported; that no important aspects of the study have been omitted; and that any discrepancies from the study as planned (and, if relevant, registered) have been explained.
