# Supplementary material for: Refugee migration and risk of schizophrenia and other non-affective psychoses: cohort study of 1.3 million people in Sweden
Source: BMJ. 2016 Mar 15;352:i1030. doi: 10.1136/bmj.i1030 (PMC4793153; doi:10.1136/bmj.i1030)
Supplement: Supplementary file 1 — Supplementary tables [file hola029606.ww1_default.pdf]

Supplementary Table A: Region of origin classification and basic sample characteristics

Region of origin[1]	SMA classification[2]	Cohort characteristics		Countries[3]
		Cases (%)	PYAR (%)	
Sweden	Sweden	3 233 (87.3)	8 385 059 (93.9)	Sweden
Sub-Saharan Africa	West Africa	20 (0.5)	10 261 (0.1)	Benin, Burkina Faso, Cape Verde, Gambia, Ghana, Guinea, Guinea Bissau, Ivory Coast, Liberia, Mali, Mauritania, Niger, Nigeria, Senegal, Sierra Leone, Togo
	East Africa	93 (2.5)	50 562 (0.6)	Djibouti, Eritrea, Ethiopia ,Somalia
	Africa, other	29 (0.8)	17 333 (0.2)	Angola, Botswana, Burundi, Cameroon, Central African Republic, Chad, Comoros, Congo, Democratic Republic of Congo, Equatorial Guinea, Gabon, Kenya, Lesotho, Madagascar, Malawi, Mauritius, Mozambique, Namibia, Rwanda, Sao Tome and Principe, Seychelles, South Africa, Swaziland, Tanzania, Uganda, Zambia, Zanzibar
Asia	Central Asia	42 (1.1)	57 020 (0.6)	Afghanistan, Armenia, Azerbaijan, Bangladesh, Bhutan, Georgia, India, Kazakhstan, Kyrgyzstan, Maldives, Nepal, Pakistan, Sri Lanka, Tajikistan, Turkmenistan
	Northeast Asia	14 (0.4)	22 714 (0.3)	China, Japan, Mongolia, People's Republic of Korea, South Korea, Taiwan
	Southeast Asia	25 (0.7)	36 946 (0.4)	Brunei, Cambodia, East Timor, Hong Kong, Indonesia, Laos, Malaysia, Myanmar, Philippines, Singapore, Thailand, Vietnam
Eastern Europe & Russia	Eastern Europe	36 (1.0)	52 957 (0.6)	Albania, Belarus, Bulgaria, Czech Republic, Hungary, Moldova, Poland, Romania, Slovakia, Ukraine
	Former Yugoslavia	36 (1.0)	58 534 (0.7)	Bosnia Herzegovina, Croatia, Kosovo, Macedonia, Montenegro, Serbia, Slovenia
	Russia & the Baltic States	15 (0.4)	29 297 (0.3)	Estonia, Latvia, Lithuania, Russia
Middle East & North African	Iran	105 (2.8)	138 638 (1.6)	Iran
	Iraq	21 (0.6)	20 057 (0.2)	Iraq
	Middle East, other	30 (0.8)	42 253 (0.5)	Bahrain, Cyprus, Israel, Jordan, Kuwait, Lebanon, Oman, Palestine, Qatar, Saudi Arabia, Syria, United Arab Emirates, Yemen, Turkey
	North Africa	6 (0.2)	6 752 (0.1)	Algeria, Egypt, Libya, Morocco, Tunisia

SMA: Swedish Migration Agency; PYAR: Person-years at-risk

[1]Predefined by authors for analysis, based on SMA classification

[2]Categories provided by the Swedish Migration Agency for research purposes, reflecting major migrant and refugee flows to Sweden

[3]Countries of origin, as defined by the Swedish Migration Agency, in each SMA category. Country-level data is not made available by the SMA/Statistics Sweden for research purposes.

Supplementary Table B: Risk of non-affective psychoses by region of origin for all immigrant groups

	**All**		**Men**		**Women**	
**Category**	**Model 1**	**Model 2**	**Model 1**	**Model 2**	**Model 1**	**Model 2**
	**HR (95% CI)**	**HR (95% CI)**	**HR (95% CI)**	**HR (95% CI)**	**HR (95% CI)**	**HR (95% CI)**
Sweden	1	1	1	1	1	1
Sub-Saharan Africa	5.24 (4.32 to 6.34)	4.10 (3.38 to 4.98)	6.68 (5.33 to 8.37)	5.25 (4.18 to 6.59)	3.64 (2.68 to 4.94)	2.82 (2.07 to 3.83)
Asia	1.96 (1.54 to 2.51)	1.50 (1.17 to 1.93)	2.04 (1.50 to 2.77)	1.55 (1.13 to 2.12)	1.88 (1.31 to 2.69)	1.43 (1.00 to 2.06)
Eastern Europe	1.74 (1.38 to 2.20)	1.41 (1.11 to 1.78)	1.74 (1.28 to 2.37)	1.42 (1.04 to 1.93)	1.75 (1.26 to 2.41)	1.38 (1.00 to 1.92)
Middle East & North Africa	2.16 (1.81 to 2.58)	1.62 (1.35 to 1.94)	2.64 (2.15 to 3.25)	2.01 (1.63 to 2.48)	1.55 (1.16 to 2.07)	1.13 (0.84 to 1.52)

Legend: HR: Hazard Ratio; 95% CI: 95% confidence interval. Model 1 was adjusted for age-at-risk, sex and their interaction (where appropriate). Model 2 was additionally adjusted for disposable income and population density. Likelihood ratio tests, on four degrees of freedom, confirmed statistical interaction between age-at-risk and sex on the risk of non-affective psychotic disorder in Model 1 (χ^2: 72.2; p<0.001) and Model 2 (χ^2: 73.6; p<0.001). LRTs, on four degrees of freedom, also confirmed statistical interaction between sex and region of origin on psychosis risk in Model 1 (χ^2: 20.7; p<0.001) and Model 2 (χ^2: 22.9; p<0.001).

Supplementary Table C: Sensitivity analysis of psychosis risk by exposure status, excluding refugee and other migrants who were diagnosed with a non-affective psychotic disorder within 12 months of arrival in Sweden

			All		Men		Women	
	**Category**	**N (excluded N, %)**	**Model 1**	**Model 2**	**Model 1**	**Model 2**	**Model 1**	**Model 2**
			**HR (95% CI)**	**HR (95% CI)**	**HR (95% CI)**	**HR (95% CI)**	**HR (95% CI)**	**HR (95% CI)**
**Refugee status**	Swedish-born	3 232 (0, 0.0)	1	1	1	1	1	1
	Non-refugee	288 (91, 24.0)	2.08 (1.79 to 2.41)	1.57 (1.34 to 1.83)	2.32 (1.94 to 2.77)	1.75 (1.46 to 2.11)	1.81 (1.47 to 2.24)	1.35 (1.09 to 1.67)
	Refugee	63 (30, 32.2)	3.10 (2.38 to 4.05)	2.44 (1.87 to 3.19)	3.74 (2.73 to 5.12)	2.98 (2.18 to 4.08)	2.21 (1.38 to 3.54)	1.69 (1.05 to 2.71)
	Non-refugee	288 (91, 24.0)	1	1	1	1	1	1
	Refugee	63 (30, 32.2)	1.49 (1.14 to 1.96)	1.56 (1.19 to 2.05)	1.61 (1.16 to 2.24)	1.70 (1.22 to 2.36)	1.22 (0.74 to 2.00)	1.25 (0.76 to 2.06)
**Region of origin**	Sweden	3 232 (0, 0.0)	1	1	1	1	1	1
	Sub-Saharan Africa	104 (38, 26.8)	4.79 (3.87 to 5.95)	3.67 (2.95 to 4.56)	6.10 (4.72 to 7.87)	4.69 (3.62 to 6.06)	3.32 (2.34 to 4.72)	2.51 (1.76 to 3.58)
	Asia	59 (22, 27.2)	1.83 (1.39 to 2.43)	1.39 (1.05 to 1.84)	1.71 (1.18 to 2.48)	1.29 (0.88 to 1.88)	2.00 (1.36 to 2.95)	1.51 (1.02 to 2.23)
	Eastern Europe	67 (20, 23.0)	1.63 (1.26 to 2.11)	1.30 (1.00 to 1.69)	1.73 (1.24 to 2.42)	1.39 (1.00 to 1.95)	1.51 (1.03 to 2.20)	1.18 (0.81 to 1.74)
	Middle East & North Africa	85 (41, 32.5)	1.89 (1.55 to 2.30)	1.38 (1.13 to 1.69)	2.21 (1.74 to 2.81)	1.64 (1.29 to 2.09)	1.47 (1.07 to 2.02)	1.05 (0.76 to 1.45)

Legend: HR: Hazard Ratio; 95% CI: 95% confidence interval. Model 1 was adjusted for age-at-risk, sex and their interaction (where appropriate). Model 2 was additionally adjusted for disposable income and population density. LRT χ^2 p-values, on two degrees of freedom, for interaction between sex and refugee status, were χ^2: 7.2; p=0.03 (Model 1) and χ^2: 8.4; p=0.02 (Model 2) and, on four degrees of freedom, between sex and region of origin, were χ^2: 13.5; p=0.01 (Model 1) and χ^2: 14.9; p=0.005 (Model 2).

Supplemental Table D: Sensitivity analysis of risk of non-affective psychoses in refugees relative to non-refugees for men by region of origin, excluding immigrants who were diagnosed within 12 months of arrival in Sweden

	**All (Model 2)**	**Men (Model 2)**
**Refugee vs non-refugee**	HR (95% CI)	HR (95% CI)
Sub-Saharan Africa	0.93 (0.55 to 1.59)	0.91 (0.46 to 1.82)
Asia	1.87 (0.96 to 3.63)	2.53 (1.12 to 5.71)
Eastern Europe	1.69 (0.73 to 3.93)	2.46 (0.96 to 6.33)
Middle East & North Africa	1.58 (1.04 to 2.41)	1.73 (1.06 to 2.85)

Legend: HR: Hazard Ratio; 95% CI: 95% confidence interval. All models were conducted on a restricted cohort, excluding the Swedish-born population and refugee or migrant cases presenting within 12 months of arrival in Sweden. Baseline groups are non-refugees from each country of origin. For all people, Model 2 was adjusted for age-at-risk, sex, their interaction, disposable income and population density. For men, it was adjusted for age-at-risk, disposable income and population density. LRT χ^2 p-values, on three degrees of freedom, for interaction between refugee status and region of origin, were χ^2: 3.7; p=0.29 (all people) and χ^2: 4.9; p=0.18 (men). Given the small sample size for women, no attempt to analyse whether hazard ratios by refugee status differed by region of origin.
